# Fueling the Future: The Emergence of Self-Powered Enzymatic Biofuel Cell Biosensors

**DOI:** 10.3390/bios14070316

**Published:** 2024-06-24

**Authors:** Akhilesh Kumar Gupta, Alexey Viktorovich Krasnoslobodtsev

**Affiliations:** Department of Physics, University of Nebraska Omaha, Omaha, NE 68182, USA; akhileshkumargupta@unomaha.edu

**Keywords:** self-powered biosensors, enzymes, bio-analytes, biofuel cell, multiplexed sensor, wearables

## Abstract

Self-powered biosensors are innovative devices that can detect and analyze biological or chemical substances without the need for an external power source. These biosensors can convert energy from the surrounding environment or the analyte itself into electrical signals for sensing and data transmission. The self-powered nature of these biosensors offers several advantages, such as portability, autonomy, and reduced waste generation from disposable batteries. They find applications in various fields, including healthcare, environmental monitoring, food safety, and wearable devices. While self-powered biosensors are a promising technology, there are still challenges to address, such as improving energy efficiency, sensitivity, and stability to make them more practical and widely adopted. This review article focuses on exploring the evolving trends in self-powered biosensor design, outlining potential advantages and limitations. With a focal point on enzymatic biofuel cell power generation, this article describes various sensing mechanisms that employ the analyte as substrate or fuel for the biocatalyst’s ability to generate current. Technical aspects of biofuel cells are also examined. Research and development in the field of self-powered biosensors is ongoing, and this review describes promising areas for further exploration within the field, identifying underexplored areas that could benefit from further investigation.

## 1. Introduction

Self-powered biosensors represent a cutting-edge innovation, enabling the detection and analysis of biological or chemical substances across diverse applications, all without reliance on external power sources [1]. These self-powered biosensors harness energy from their surroundings or even from the analyte itself, efficiently transforming it into electrical signals for precise sensing and seamless data transmission [2]. The inherent self-sufficiency of these biosensors delivers a range of compelling benefits, including portability, operational autonomy, and a significant reduction in waste stemming from disposable batteries. Their far-reaching utility spans many domains, including the following important sectors: Healthcare, environmental monitoring, and food safety [3,4].

Self-powered biosensors commonly employ energy harvesting techniques to transform environmental energy sources like body heat, motion, or sunlight into usable power for sensor functionality [5]. Biosensors are categorized based on their power sources. These general categories most often include the devices harvesting the following energies: (1) piezoelectric energy—generates electricity when subjected to mechanical stress or vibrations; (2) thermoelectric energy—utilizes the temperature differences between the sensor and its environment to generate electricity; (3) triboelectric energy—generate electricity through friction; (4) photovoltaic cells—biosensors that use photovoltaic materials to convert light energy into electricity; and (5) biofuel cells (BFCs)—employ microbial cells or enzymes to catalyze the oxidation of organic compounds present in the sample. The oxidation reaction releases electrons, which can be harvested as electrical energy to power the sensor [6]. Biofuel cells can serve not only as power sources for sensing devices but also as sensors themselves [7]. This dual functionality is possible when the enzyme and its concentration on the electrode are carefully selected to match the concentrations of the species being measured [8]. By optimizing these parameters, biofuel cells can generate an electrical signal in response to specific biochemical reactions, allowing them to both power and detect the presence of target analytes [9].

The area of self-powered biosensors has been rapidly evolving over recent years [10]. The evolution of these biosensors is closely tied to the new discoveries in nanoengineering and nanomaterial innovation. Mainly, the improvements seek to enhance the sensor’s performance while addressing sensitivity and selectivity concerns. The convergence of self-powered biosensors with wearable technology represents a promising development in healthcare, wellness, and environmental monitoring [11]. Although self-powered biosensors are an exciting area of research and development, they hold several challenges that need to be addressed to make them more practical and effective. Some of the key challenges include (1) sensitivity to reach a biologically relevant range of analyte concentration, (2) selectivity to discern among various analytes, (3) robustness, and (4) miniature nature. Ongoing research and advancements in materials science, nanotechnology, and energy harvesting techniques are steadily improving self-powered biosensor technology [12]. Many recent studies also focused on enhancing the efficiency of the cathode reaction, which, in many cases, is the main limitation of biofuel cell design, especially for powering medical implantable devices [9,13]. As these challenges are addressed, self-powered biosensors hold tremendous potential for transforming the fields of healthcare, environmental monitoring, food safety, and others [14]. This review focuses on biofuel cells that use enzymes to initiate electron transport via organic molecules as fuel and future challenges towards next-generation applications. Biofuel cells offer a combination of sustainability, miniaturization, biocompatibility, real-time monitoring, self-powered operation, environmental friendliness, and cost-effectiveness. These characteristics make them an excellent choice for powering self-sufficient biosensors across a range of applications.

## 2. Biofuel Cells as Energy Source for Self-Powered Biosensors

**Figure 1:**
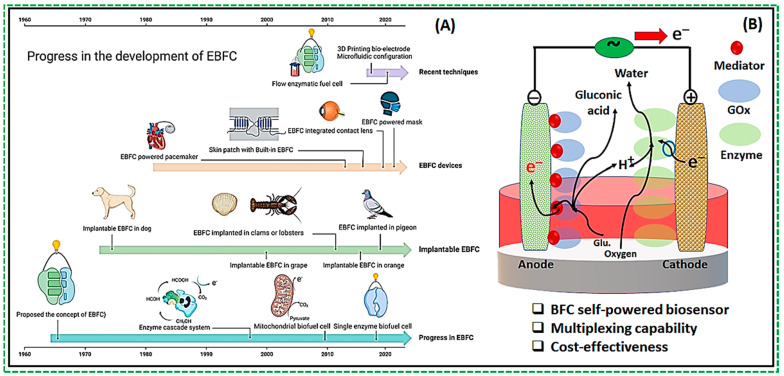
(**A**) Progress in the development of enzymatic biofuel cells (EBFC) and applications [27]; (**B**) Schematic of a design of the enzymatic fuel cell (EFC) with both anode and cathode being bioelectrodes containing enzymes.

One of the very dynamic areas of research in harnessing energy for self-powered operation is the development of biofuel cells. Biofuel cells utilize the chemical energy produced by biological reactions to produce electrical energy [15]. These cells replicate the energy conversion mechanisms observed in living organisms, such as bacteria [16]. Biofuel cells are classified into two main categories: (1) microbial and (2) enzymatic. In both types, the key concept involves integrating biocatalysts with electrodes to facilitate the transfer of electrons between the electrode and the redox-active centers of the enzymes, whether they are isolated or contained within bacterial cells. Enzyme-based biofuel cells (EFCs) have been documented since the early 1960s [17]. However, the development of enzymatic biofuel cells has experienced significant growth in recent years, leading to improvement in their stability and power output, Figure 1A. Recent studies have focused on elucidating the reaction mechanisms of enzyme catalytic reactions [18,19], developing novel biomaterials [20], exploring enzyme modification techniques, [21] investigating enzyme immobilization methods [22,23], and refining enzyme electrode structures [9,24]. Current research efforts are focused on enhancing the performance of enzyme-modified electrodes [25]. Various enzymes can be utilized in enzyme fuel cells (EFCs), with a notable example being the development of glucose biosensors [26].

The classical design of the EBFC involves the modification of an electrode with a substrate-oxidizing enzyme within an enzymatic fuel cell (EFC). Operation of such a device depends on redox enzyme catalysis at the interface of at least one electrode, with metal electrodes traditionally performing the circuit-closing reaction (i.e., the reduction of O_2_ to H_2_O on Pt cathodes) [26]. However, redox enzymes can also be employed on cathodes to facilitate the reduction of O_2_ to H_2_O. When enzymes are applied to both the anode and cathode, the cell is termed a full Enzymatic Biofuel Cell [26]. Figure 1B illustrates a typical configuration of the full EBFC, where the anode and cathode are used as bioelectrodes. Specifically, the anode is modified with glucose oxidase (GOx), while the cathode is modified with another redox enzyme capable of reducing oxygen to water. An electrolyte separates the two bioelectrodes to complete the cell’s structure. In the example of GOx-modified cells, the enzyme immobilized on the electrode surface catalyzes glucose oxidation, producing gluconolactone through a two-electron process. The electrons are then transferred to the cathode through an external circuit, generating an electrical current.

The performance of the fuel cell is mainly dependent on the enzyme activity. One of the main challenges for EFCs is to establish efficient electron transfer between enzymes and electrode supports. Fuel cell biosensors are categorized into two types based on how charge transfer occurs between the catalytic site and an electrode: (1) direct charge transfer (DET), 1st and 3rd generation of EFCs, and (2) indirect or mediated charge transfer (MET), 2nd generation of EFCs [24].

Although enzymatic biofuel cells have several advantages, their limited power output is largely due to inefficient charge transfer coupling between the electrode interface and the immobilized enzyme. Advanced biosensors rely on mediated electron transfer (MET), where redox mediators are employed (2nd generation). These mediators are small electroactive molecules that shuttle electrons between the enzyme’s active sites and an electrode. They can exist as either freely diffusing mediators or be bound to the side chains of flexible redox polymers. Recent innovative designs of electrodes have introduced effective charge mediators, which enhance the performance of biofuel cells by substantially increasing the electron transfer kinetics [28]. For example, an assembly of amphiphilic layers composed of glucose oxidases in aqueous media and hydrophobic/conductive nanoparticles in nonpolar media deposited onto cotton fiber/textile to construct the anode notably increases electron transfer efficiency [29].

Direct charge transfer types include biosensors where either the products of enzymatic reaction are utilized at the electrode (1st generation) or a direct electronic connection between the enzyme’s redox center and the electrode surface is established (3rd generation). The third generation of DET biosensors certainly simplifies the design of the cells, contributing to their robustness. A comprehensive search for enzymes capable of direct electron transfer is currently underway, with several already reported from single-cofactor, multi-cofactor, and fusion enzyme families [30]. Heme-containing enzymes such as peroxidases are notably promising catalysts for biosensors owing to their high DET currents [30].

Research has focused on advancements in electrode materials, device structures, and various modification techniques to enhance the performance of BFCs [25]. Advances in new electrode materials further improve biosensor designs and enhance the coupling for electron transfer rates [31]. In recent years, significant strides have been made in developing novel electrode materials and electrolytes or modifying existing ones in electrochemical systems. These advancements are driven by the desire to attain optimal parameters during the cell’s operation, especially specific capacity, cyclic stability, and Coulombic efficiency. Hybrid materials and nanocomposites emerge as promising electrode materials, addressing the limitations of commonly used materials [32].

For example, employing high surface area nanomaterials like carbon nanotubes (CNTs), graphene, and metal–organic frameworks (MOFs) can significantly increase the electrode’s surface area, facilitating greater enzyme loading and improved electron transfer rates. Using such nanomaterials as carbon nanotubes, graphene oxide (GO), noble metal nanoparticles, and conjugated polymers (CPs) enables improvements in electron transfer efficiency and amplification of generated electrical signals and offers a stable matrix for enzyme immobilization [33]. Various processes are employed to obtain polymer nanocomposites, including mixing dispersed particles with polymers in liquids, mixing particles with monomers followed by polymerization, formation of nanocomposites using molten or solid polymers, and simultaneous formation of particles and polymers [34]. Additionally, surface modification techniques, including the immobilization of enzymes via covalent bonding, entrapment, or adsorption, can improve enzyme stability and activity [35]. Co-immobilization of redox mediators with enzymes or incorporating conductive polymers can further enhance electron transfer efficiency [36]. Collectively, these strategies aim to maximize the catalytic activity and stability of single-enzyme-based biosensors. Furthermore, optimizing device structures with the utilization of three-dimensional configurations and microfluidic channels can further enhance mass transport and minimize diffusion limitations [37].

Additional challenges faced by the field of biosensor development include their cost and durability. The production and scaling up of enzymatic or microbial fuel cells can be expensive, thus impeding their widespread adoption. Moreover, maintaining the activity of biological catalysts over time also presents a significant challenge. There are also challenges related to energy requirements and the integration of biocatalysts with the rest of the circuitry. Despite numerous advantages, full BFCs have some drawbacks. First, the use of two different enzymes complicates the design and increases their cost. Second, the optimal operating conditions for individual enzymes may vary, thereby affecting BFC’s performance. In the next section, we explore the feasibility of single-enzyme-based biofuel cells and the potential of next-generation advancements for EBFCs.

## 3. Development of a New Biocathode for a Single-Enzyme Biofuel Cell

The high specificity of enzymes to their respective substrate under mild operating conditions makes them an attractive way to build effective EBFCs. Additionally, enzymes immobilized on the electrode surface allow for membrane-less configuration of EBFCs, opening opportunities for developing miniaturized systems for self-powered biosensors and electronic devices such as insulin pumps, hearing aids, bone stimulators, and pacemakers [38].

Typically, full EBFCs employ two distinct enzymes: one to oxidize the fuel substrate at the anode and another to reduce oxygen at the cathode, Figure 1B. Following the substrate’s oxidation by the anode enzymes, hydrogen protons and electrons produced in the reaction travel through the electrolyte and external circuit, respectively, reaching the cathode, where they participate in charge transfer via reduction [27]. Currently, widely explored enzymes for these roles include glucose oxidase (GOx), lactate oxidase (LOx), hydrogenases, and glucose dehydrogenase (GDH) for the anode, while laccase and bilirubin oxidase (BOD) represent common choices for the cathode. Despite the advantages, employing two different enzymes complicates the design of EBFC systems and increases their overall cost. This challenge could potentially be addressed by developing an electrode that serves both the anodic and cathodic functions but is modified with a single enzyme.

**Figure 2:**
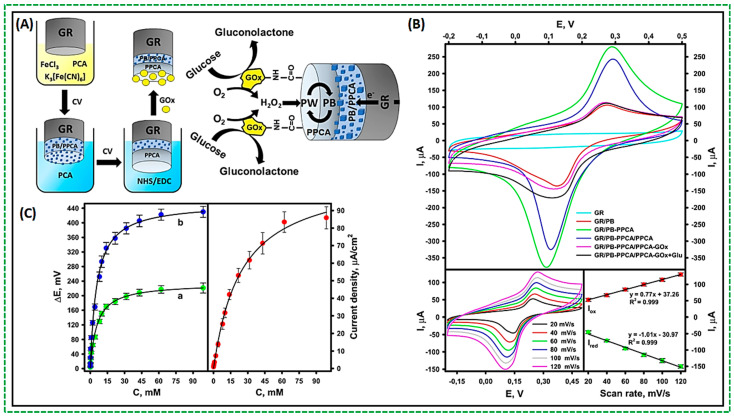
(**A**) Schematic of biocathode fabrication; (**B**) Cyclic voltammetry of fabricated electrodes and their response concerning modifications; (**C**) Dependent of generated potential concerning glucose concentration. At 98.86 mM glucose, the peak current density reached 85.86 ± 6.30 A/cm^2^, the potential was 221.03 ± 13.90 mV, and the OCP was 430.15 ± 15.10 mV [39].

A novel design has recently emerged featuring a cathode incorporating Prussian Blue (PB) as an “artificial” peroxidase (PO). PB, possessing peroxidase-like characteristics, exhibits an increased catalytic activity towards H_2_O_2_. In enzyme reactions producing H_2_O_2_, PB can effectively substitute an enzyme. Consequently, PB has received significant attention for its application in electrochemical biosensors [39,40]. Its remarkable catalytic activity redirects the reduction of H_2_O_2_ to a more positive potential than O_2_ reduction, thereby mitigating interference from oxygen and other contaminants. The mechanism underlying H_2_O_2_ reduction on PB-modified electrodes is as follows: PB undergoes electrochemical reduction, yielding Prussian White (PW), which catalyzes the reduction of H_2_O_2_ at a low potential. Subsequently, PW is oxidized back to PB. Due to its reversible electrochemical redox properties, PB serves as a renewable catalyst throughout the electrochemical process. In some recent studies, GOx was utilized in conjunction with PB as the cathode modification for the design of a single-enzyme biosensor [39,41]. First, a graphite rod (GR) was coated with a composite of PB particles embedded in the poly(pyrrole-2-carboxylic acid) (PPCA) shell (GR/PB-PPCA), and an additional layer of PPCA (GR/PB-PPCA/PPCA) by cyclic voltammetry (CV). Another PPCA layer (GR/PB-PPCA/PPCA) was deposited as a protection layer for GOx. Amide bonds formed between GOx molecules and PPCA carboxyl groups activated by a combination of EDC and NHS. Figure 2A depicts the design concept and operation of the hybrid biocathode. The operation of the biocathode can be described as a cascade of redox reactions driven by the electrocatalytic activity of PB towards the reduction of H_2_O_2_ formed during GOx-catalyzed oxidation of glucose. After receiving the electron, Fe (III) of PB is electrochemically reduced to form PW, which has a high reduction activity. PW reduces the H_2_O_2_ formed during enzymatic reaction, and the PW is re-oxidized to PB. The cyclic voltammograms show a pair of redox peaks attributed to the electrochemical reaction of ferric ions in PB (Fe^2+^/Fe^3+^ transition), Figure 2B. The presence of typical redox peaks and an increase in peak current compared to the GR electrode show that PB was successfully deposited on all electrodes. The uniform dispersion of PB inside the polymer matrix provides smooth electrode operation. Such a design responds nicely to the increased glucose concentrations, as depicted in Figure 2C. The increasing changes in current and potential in response to various glucose concentrations are easily identifiable and interpretable. As can be seen, increases in the glucose concentration up to 10 mM resulted in a linear signal increase. A non-linear increment in the biocathode response was found with increasing glucose concentration up to 98.86 mM, after which the recorded signal became saturated because the catalytic reaction was blocked at higher glucose concentrations [39].

Our group also fabricated a novel design utilizing a single-enzyme biocathode within the biofuel cell powered by glucose [42]. We modified a working electrode in the three-electrode setup of the screen-printed electrode with AgNCs@DNA [43] combined with poly (pyrrole-2-carboxylic acid)-PPCA and GOx [42]. We based our design on the reversible activity of AgNCs@DNA, which are oxidized by H_2_O_2_ and get reduced electrochemically during the continuous CV cycle. 

In this design, AgNCs@DNA effectively replaces the requirement for the second cathodic enzyme, acting as an “artificial” peroxidase similar to PB [44]. In our design, GOx enzymes are covalently attached to the top of the PPCA polymer layer, while AgNCs@DNA are embedded within the PPCA layer [43]. This allows them to react promptly with the H_2_O_2_ produced when GOx quickly processes glucose molecules at the outer surface of the electrode. Polymer-embedded AgNCs act as charge transport mediators, successfully transporting charges from the GOx-modified outer layer to the electrode surface. The benefit of such biofuel cells is the simplified system without the need for two distinct enzymes. Table 1 compares several recently reported single-enzyme EBCs, including both PB and other non-PB designs.

While PB is widely employed as a very well-working substance with “artificial peroxidase” activity that also participates in electron transfer [46], others can also be used. Examples include carbon-based materials such as CNT, MWCNT, graphene oxide, graphite, and conducting polymers, such as PEDOT:PSS [53,54]. 

## 4. Applications of Enzymatic Biofuel Cell Design for Implantable and Wearable Self-Powered Biosensors

**Figure 3:**
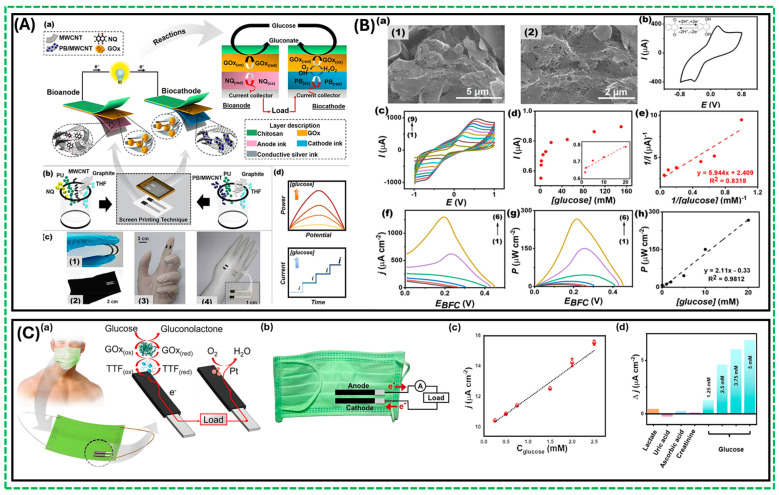
(**A**) (a–c) Schematic diagram and reactions involved due to electron transport between the GOx active site and the NQ-mediated nanocomposite; (**B**) (a) SEM morphology showing the composition of MWCNTs and graphite particles dispersed uniformly; (b) CV response of screen-printed NQ/MWCNT-based anode, pH 7.0 at a scan rate of 50 mV s^−1^; (c) CVs response of a screen-printed NQ/MWCNT-based anode, pH 7.0 at different scan rates; (d) The corresponding calibration plot of the current response of the GOx/NQ/MWCNT-based bioanode; (e) The double reciprocal plot of the calibration curve obtained from the GOx/NQ/MWCNT-based bioanode (f–g) Polarization curves, and power density versus potential plots of a screen-printed glucose BFC at different glucose concentrations as ((1) blank; (2) 1 mM; (3) 2 mM; (4) 5 mM; (5) 10 mM; and (6) 20 mM) in 0.1 M PBS, pH 7.0; (h) The corresponding calibration plot of power and glucose concentrations [50]; (**C**) (a) The concept of the mask-based bioelectronic device using glucose-containing sweat as fuel to produce electricity for self-powered operation; (b) The components of the mask-based bioelectronic device anode and the cathode; (c) The self-powered current output is achieved when glucose concentrations are increased; (d) Self-powered responses toward glucose and other substances [56].

A single-enzyme biocathode biosensor design minimizes cost and reduces demands for operating conditions. More importantly, this design uses glucose as the sole fuel source, and its generated current density and open circuit potential (OCP) are dependent on glucose concentration [39,42]. Moreover, the byproduct of the operation, gluconolactone, is biocompatible, making it possible to use as the power source in implantable devices. Recently, Veenuttranon et al. fabricated screen-printable nanocomposite inks, single-enzyme-based energy harvesting devices for self-powered biosensing systems [50]. This first screen-printable prototype was a flexible self-powered biosensor that consumed only glucose as fuel and exhibited compatibility with various substrates, including flexible textiles, plastic, stretchable epidermal tattoos, and rubber-based materials. Figure 3A demonstrates (a) the basics of the operation of such a device and the enzymatic reactions involved, (b) the preparation of the screen-printable inks for the anode and the cathode, (c) screen-printed glucose BFC at different substrates and capabilities, and (d) the resulting current and power generation which are directly proportional to the glucose concentration. In addition, a single enzyme, GOx, is used on both bioanode and biocathode; the two electrodes utilize different charge mediators. Biocathode utilized Prussian blue (PB) as an electrocatalyst due to its activity towards H_2_O_2_. The bioanode utilized naphthoquinone (NQ) as the effective charge mediator between GOx and the surface of the electrode. Additional improvements to the cell’s performance can be introduced by increasing the surface area of the electrode, which was achieved by dispersing MWCNT/graphite particles at the anode. This structure with high porosity improved electrochemical characteristics and incorporated the embedment of NQ molecules in the matrix, as shown in Figure 3B(a). Figure 3B(b) illustrates that NQ is embedded in the printed matrix and displays a quasi-reversible redox capability. Moreover, Figure 3B(c) demonstrates that the NQ/MWCNT-based electrode performs well at various scan rates. After successful modification with GOx, this composite NQ/MWCNT-based electrode produced an anodic current, which increased with the increasing concentration of glucose, Figure 3B(d). The designed cell was deemed suitable for self-powered biosensing applications due to linear response within the calibration range, Figure 3B(e). Thus, the group developed a novel screen-printable membrane-less single-enzyme-based energy-harvesting device and a self-powered biosensor driven by the same substrate (glucose). Also, they evaluated the performance of a single-enzyme BFC, as shown in Figure 3B(f,g), which exhibited an OCP of 0.45 V with a maximum power density of 266 μWcm^−2^ and a current density of ~ 1.3 mA cm^−2^ at 20 mM glucose. Figure 3B(h) shows a good relationship between the increasing glucose concentrations and power density in a 0–20 mM glucose range, with good sensitivity (2.1 ± 0.1 μW cm^−2^ mM^−1^) and a correlation coefficient of 0.9812 [50]. Another example of hybrid design is the fabrication of a flexible reduced graphene oxide and gold nanoparticle-modified electrochemical biosensor design utilizing gel instead of liquid electrolytes and a combined stretchable nanocomposite, which exhibited high catalytic activity with specificity and selectivity towards glucose. Furthermore, using a soft gel offers additional benefits, including superior skin adhesion and enhanced permeability of water-soluble molecules, improving thus the accumulation of bodily fluids on dry skin surfaces [55].

Printing flexible electrodes significantly enhances the capacity to create flexible wearable devices. Figure 3C(a) shows a self-powered design on a mask for continuous glucose monitoring, prepared using the screen-printing technique and subsequent functionalization of catalytic layers for both the bioanode (CNT/PANI/TTF/GOx) and cathode (CNT/PEDOT/Pt) [56]. These flexible electrodes exhibit high capacitance and improved biocatalytic activity. The mask-based energy device utilizes a commercial face mask integrated with engineered biofuel cell (BFC) and supercapacitor systems, as shown in Figure 3C(b). The capacitance of the printed electrodes, which include conducting polymers and CNT, exhibits the characteristics of the electric double layer at the interface between the wearable electrode and the biofluid [57], facilitating effective charging and power generation. The BFC’s performance, tested in different glucose concentrations under pH 7.0 and ambient conditions, demonstrated remarkable stability and high performance, as shown in Figure 3C(c). The mask-based BFC provided an open circuit voltage of 0.37 V and a maximum output density of 14 μW/cm^−2^ at a voltage of around 0.25 V. Figure 3C(c) shows that the increase in glucose concentration results in a linear response of the current density for the designed device, while minor changes are observed when adding interfering species, Figure 3C(d).

In general, an approach involving a single enzyme with modifications on a single electrode design holds promise for biofuel cell-based biosensing applications, particularly when leveraging bodily fluids as a sustainable energy source for continuous operation [58]. Attractive examples of bodily fluids include sweat, saliva, tears, or interstitial fluid. Sweat, abundant on the skin and easily collectible non-invasively, stands out due to its high glucose and lactate content, rendering it compatible with previously described single-enzyme designs. However, for optimal efficiency, these biosensors must mimic skin properties and utilize effective means of sweat collection. Therefore, future developments of such devices may include material improvements with characteristics similar to human skin, such as mechanical durability, flexibility, and stretchability. Such devices are collectively termed e-skin or electronic skin devices [59]. The potential of the new era of non-invasive and wearable health monitoring applications primarily defines success in this field.

The concept of a flexible electrode is greatly enhanced by the simplicity of polymerization, whether conductive or non-conductive polymers [60]. Additional modifications, such as those described above in the design of the single-enzyme biocathode EBFC, offer clear advantages. If placed on top of a flexible carbon or silver ink [61], such electrodes become self-powered stretchable precursors for e-skin wearable sensors. Screen-printing technology can easily incorporate such a modification [62]. Additional improvements in the cell performance can be achieved by increasing surface area and other modification strategies of the electrode. For example, electrospinning can convert filament-forming polymers into nanofibers, serving as substrates for electrode manufacturing. This approach ensures efficient enzyme immobilization and has various other functions, such as catalysis and electrical conductivity, integrating polymers (substrate) and functional ingredients (i.e., conductivity, biocompatibility, and catalysis). Importantly, the stretchable electrode design establishes a stable platform of self-powered devices and holds promises for seamless integration into wearable electronics [63].

**Figure 4:**
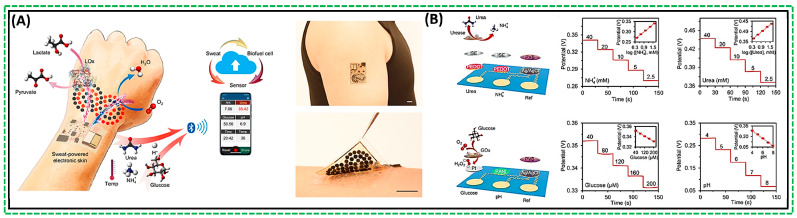
(**A**) Serpentine-connected electrode arrays act as biosensors; (**B**) Soft electrochemical patch used to fabricate NH^4+^ and urea sensor array [64].

The designs aimed at accommodating the needs for biocompatibility, flexibility, and high performance often require complexity. For example, Yu et al. recently reported a flexible, perspiration-powered integrated electronic skin (PPES) for in situ metabolic sensing [64]. The fabricated flexible electrochemical patch featured a nanoengineered configuration comprising serpentine-connected electrode arrays of biosensing films and biocatalytic nanomaterials, as illustrated in Figure 4A [64]. Lactate oxidase (LOx) immobilized on bioanodes facilitated stable current. The bioanode was a modified hierarchical Ni (h-Ni) microstructure with reduced graphene oxide (rGO) films and Meldola’s blue–tetrathiafulvalene–modified carbon nanotubes (MDB-TTF-CNT) on an Au electrode array. In addition, the flexible patch was consolidated with rigid electronics on an ultrathin polyimide (PI) substrate for power management, signal processing, and wireless transmission [64]. This battery-free e-skin sensing system was a highly efficient lactate biofuel cell that offered multiplexed sensing capability for various sweat components, revealing crucial physiological conditions that can serve as biomarkers [65]. The incorporation of multiplexed sensing is invaluable for accurately assessing individual analytes [65]. Therefore, the soft electrochemical patch with NH^4+^ ion-selective electrodes was used to construct the NH^4+^ and urea sensor array, as shown in Figure 4B [64]. A linear relationship was found between the target analytes’ potential output and logarithmic concentrations, suggesting a good-performing biosensor.

Huang et al. reported the simultaneous detection of lactate and glucose in sweat in real-time, and they described a stretchable self-powered biosensor with an epidermal electronic format [66]. The enzymatic biofuel cell, featuring bioanode modifications with GOx and LOx, served as a self-powered sensing module enabling the perspiration sensor. Impressively, the sensor exhibited a sensitivity of 2.48 mV/mM for lactate and 0.11 mV/M for glucose detection. To accommodate this functionality, a multilayered design of electrodes was necessary, integrating a graphene layer and a microfluidic module fabricated via photolithography and PDMS molding. Notably, a linear relationship between the open circuit potential (OCP), lactate, and glucose concentration was produced, confirming the reliable performance of the sensor. These epidermal self-powered sweat sensors were tested for in situ and real-time sweat analysis on various body parts, including the forehead, chest, and back [66].

The development of enzymatic biofuel cells and their applications in wearable and implantable devices has experienced significant growth in recent years [67]. EBFCs have been utilized as power sources for various applications, including self-powered ingestible devices for real-time monitoring of metabolites [68], stent integration [69], incorporation into artificial veins [70], and brain stimulation in birds [71]. These are just a few examples of recent successes in the development of implantable devices [72]. Implantable and wearable devices are playing an increasingly significant role in modern healthcare, improving patient outcomes, enhancing quality of life, and advancing medical research and technology.

## 5. Conclusions, Challenges, and Prospects

Self-powered biosensors based on enzyme–biofuel cells can provide exceptional selectivity, sensitivity, and an almost immediate response time. They hold promises across diverse practical fields: (1) Biomedical applications: EBFCs are being explored for use in medical devices, where they can potentially power these devices using organic substrates such as glucose or lactate from bodily fluids. Examples include wearable and implantable devices, which could lead to more effective and less invasive healthcare solutions, improving patient outcomes and enhancing quality of life. (2) Wearable and implantable bioelectronics: EBFCs have the potential to power small electronic devices, for example, sensors and wearables, by utilizing readily available bodily fluids such as sweat. These innovative solutions could lead to more effective and less invasive healthcare solutions, improving patient outcomes and enhancing quality of life. (3) Remote sensors: EBFCs can be used to power sensors in remote or resource-limited locations where conventional power sources are impractical. This application is particularly valuable for environmental monitoring, agricultural applications, and in areas with limited access to electricity, enabling reliable data collection and transmission in challenging conditions.

Despite their potential, several challenges remain in the field of EBFCs, including (A) cost reduction by making the technology more affordable for widespread adoption; (B) improving performance by enhancing the power output and efficiency of the cells to meet the demands of various applications; (C) device stability by ensuring the long-term stability and reliability of EBFCs in different environments; and (D) biocompatibility by developing materials and designs that are compatible with biological systems, minimizing any adverse reactions.

Ongoing research explores various promising directions to address these challenges, such as novel simplified cell design by creating more efficient and straightforward designs that reduce complexity and cost; advanced material fabrication by developing new materials that improve the efficiency and durability of EBFCs; and introduction of novel redox enzymes by utilizing innovative enzymes that enhance the catalytic efficiency and stability of the biofuel cells.

The emergence of self-powered biosensors based on EBFCs presents significant potential for fueling the future of healthcare solutions. By powering the next generation of biomedical devices, EBFCs could transform patient care, enable more effective monitoring and treatment, and drive advancements in medical research and technology. As these technologies continue to evolve, they promise to play an increasingly critical role in modern healthcare, enhancing the quality of life and outcomes for patients worldwide.

## Figures and Tables

**Table 1:** Summary of recent studies involving single-enzyme biosensor design.

Material/Electrode	Sensitivity, µA/mM	LOD, mM	Power Density	Ref.
GRE/PB-PPCA/PPCA–GOx	0.16	0.07	-	[45]
GRE/PPD/(AuNPs)PPCA–GOx	-	-	10.94 μW/cm^2^	[46]
GR/PB-PPCA/PPCA–GOx	-	-	0.35 μW/cm^2^	[39]
GR/PPD/(AuNP)PPCA/GOx, GR/PPD/PPCA/GOx	0.135	0.08	-	[47]
BC/c-MWCNTs/ZIF-8/LAC	-	1.95 × 10^−3^	3.68 W/m^3^	[48]
laccase–CNT	-	-	140 mW/m^3^	[49]
GOx/NQ/MWCNT	0.4738	-	266 μW/cm^2^	[50]
AgNCs–PPCA/PPCA–GOx	-	2.3 × 10^−2^	4 mW/cm^2^	[42]
AOBC/c-MWCNTs-LAC	-	-	1.897 W/m^3^	[51]
PU/ZIF-8@LAC/CNTs	-	-	1.33 W/m^3^	[52]
